# Early-life inhalant allergen exposure, filaggrin genotype, and the development of sensitization from infancy to adolescence

**DOI:** 10.1016/j.jaci.2019.08.041

**Published:** 2020-03

**Authors:** Angela Simpson, Helen A. Brough, Sadia Haider, Danielle Belgrave, Clare S. Murray, Adnan Custovic

**Affiliations:** a Division of Infection, Immunity and Respiratory Medicine, Faculty of Biology, Medicine and Health, Manchester Academic Health Sciences Centre, University of Manchester and University Hospital of South Manchester NHS Foundation Trust, Manchester, United Kingdom; b Children's Allergy Service, Evelina London, Guys and St Thomas' NHS Trust, London, United Kingdom; c Paediatric Allergy Group, Department of Women and Children's Heath, School of Life Course Sciences, London, United Kingdom; d Paediatric Allergy Group, School of Immunology & Microbial Sciences, King's College London, London, United Kingdom; e Section of Paediatrics, Imperial College London, United Kingdom; f National Heart and Lung Institute, Imperial College London, United Kingdom; g Microsoft Research Cambridge, Cambridge, United Kingdom

**Keywords:** Allergen exposure, house dust mite, cat, dog, sensitization, birth cohort, filaggrin, childhood, Fel d 1, Der p 1, Can f 1, AD, Atopic dermatitis, FLG, Filaggrin, GEE, Generalized estimating equations, HDM, House dust mite, OR, Odds ratio

## Abstract

**Background:**

We hypothesized that filaggrin *(FLG)* loss-of-function mutations modify the effect of allergen exposure on the development of allergic sensitization.

**Objective:**

We sought to determine whether early-life exposure to inhalant allergens increases the risk of specific sensitization and whether *FLG* mutations modulate these odds.

**Methods:**

In a population-based birth cohort we measured mite, cat, and dog allergen levels in dust samples collected from homes within the first year of life. Sensitization was assessed at 6 time points between infancy and age 16 years. Genotyping was performed for 6 *FLG* mutations.

**Results:**

In the longitudinal multivariable model (age 1-16 years), we observed a significant interaction between *FLG* and Fel d 1 exposure on cat sensitization, with the effect of exposure being significantly greater among children with *FLG* mutations compared with those without (odds ratio, 1.36; 95% CI, 1.02-1.80; *P* = .035). The increase in risk of mite sensitization with increasing Der p 1 exposure was consistently greater among children with *FLG* mutations, but the interaction did not reach statistical significance. Different associations were observed for dogs: there was a significant interaction between *FLG* and dog ownership, but the risk of sensitization to any allergen was significantly lower among children with *FLG* mutations who were exposed to a dog in infancy (odds ratio, 0.16; 95% CI, 0.03-0.86; *P* = .03).

**Conclusions:**

*FLG* loss-of-function mutations modify the relationship between allergen exposure and sensitization, but effects differ at different ages and between different allergens.

## Graphical abstract

**Figure:**
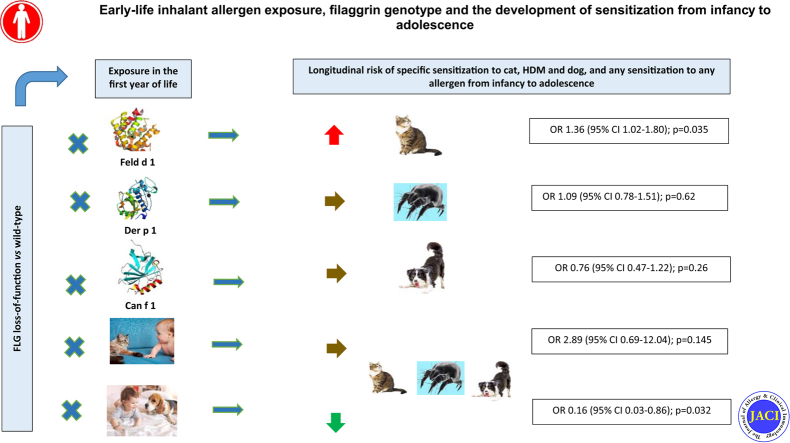

Although being exposed to an allergen or allergens is a prerequisite for the development of sensitization, the nature of the relationship between the level of exposure and the risk of sensitization is unclear.^1^^,^^2^ For example, in some studies exposure to house dust mite (HDM) allergens has been shown to increase the risk of HDM sensitization and asthma,^3^, ^4^, ^5^, ^6^ particularly in children with parental atopy.^7^^,^^8^ However, other studies have not confirmed this association (reviewed by Custovic^1^).^9^ Similarly, conflicting data have been reported on the effect of cat ownership and Fel d 1 exposure on cat sensitization, which have been shown in different studies to be either risk^6^^,^^9^, ^10^, ^11^ or protective^12^^,^^13^ factors. Early-life exposure to dogs in the home has been shown to reduce subsequent risk of allergic sensitization to multiple allergens,^14^ but no studies have assessed the effect of objectively measured dog allergen levels in homes on specific sensitization.

A recent study has reported different associations between early-life cat exposure and sensitization to cat at different ages in a birth cohort, pointing to the importance of life-course perspective.^15^ In the first 3 years of life, sensitization was more common among cat owners, but after this, the increase in sensitization rate was greater among children without a cat, and therefore by adolescence, the prevalence of sensitization was numerically greater in this group (although the difference was not statistically significant).^15^ Hence apparently contradictory results might be a consequence of different life-course sensitization trajectories between exposed and nonexposed subjects. Therefore, to understand the complex relationship between early-life exposures and later clinical outcomes, one should not rely only on cross-sectional analyses because more useful information can be gained through analysis of longitudinal trajectories.^1^^,^^16^

The effect of early allergen exposure on sensitization is modified by parental atopy and birth order,^17^ alluding to the importance of both genetic and environmental factors.^1^ The concept that the same environmental exposure can have different effects among subjects with different genetic predisposition has been tested in studies that assessed the interaction between genes and the susceptibility to environmental factors.^18^, ^19^, ^20^ Variability in response to HDM exposure in relation to mite-specific sensitization has been attributed to the *IL4* gene promoter polymorphism C-590T.^21^ Filaggrin *(FLG)* loss-of-function mutations contribute to an impaired skin barrier and are associated with eczema and a range of allergic conditions,^22^, ^23^, ^24^ as well as allergic sensitization.^24^^,^^25^ Children with *FLG* mutations were found to have an increased risk of eczema if they were exposed to cat in early life, with no effect of exposure among those without *FLG* mutations.^26^

In a study on food allergy, we have shown that early-life exposure to peanut allergens measured in dust collected from homes is associated with an increased risk of peanut sensitization and allergy in children who carry FLG mutations, with no significant effect of exposure in those without *FLG* mutations.^27^ In the current study we hypothesized that *FLG* loss-of-function mutations would modify the effect of exposure to inhalant allergen (HDM, cat, and dog) on the development of sensitization. To test this, we used both cross-sectional and longitudinal analyses to investigate the effect of early-life domestic allergen exposure on subsequent sensitization and whether these relationships were altered by *FLG* genotype and modified over time.

## Methods

### Study design, setting, participants, data sources, and definition of outcomes

The Manchester Asthma and Allergy Study is an unselected birth cohort described in detail elsewhere.^28^ A detailed description is provided in the Methods section in this article’s Online Repository at www.jacionline.org. Briefly, 1184 subjects were recruited prenatally and followed prospectively. For this study, we used data from 1051 children in the observational cohort, excluding 133 children who took part in the environmental intervention arm.^29^^,^^30^ The study was approved by the local ethics committee, and parents provided written informed consent.

Participants attended follow-up visits at ages 1, 3, 5, 8, 11, and 16 years. We assessed sensitization by skin prick tests. Mite, cat, and dog sensitization were defined as a wheal diameter at least 3 mm larger than that elicited by the negative control. Allergic sensitization was defined as a positive skin prick test response to at least 1 of the allergens tested. Cat and dog ownership in the first year of life was ascertained by using questionnaires administered at home visit in the first year of life.

### Quantification of allergen exposure

Dust samples were collected in the first year of life from the living room and child’s bedroom floors.^31^ Der p 1, Fel d 1, and Can f 1 levels were measured by using an mAb-based ELISA (Indoor Biotechnologies, Cardiff, United Kingdom) with a detection limit of 0.2 μg/g, as previously described.^32^^,^^33^ To determine an individual child’s allergen exposure, we averaged allergen concentrations in samples taken from the living room and child’s bedroom.

### Genotyping

*FLG* genotyping was performed by using probes and primers, as previously described.^26^^,^^27^^,^^34^ Genotyping for R501X, S3247X, and R2447X mutations was performed with a TaqMan-based allelic discrimination assay (Applied Biosystems, Cheshire, United Kingdom). Mutation 2282del4 was genotyped by sizing of a fluorescently labeled PCR fragment on a 3100 or 3730 DNA sequencer. *FLG* mutations 3673delC and 3702delG were assessed by using GeneScan analysis of fluorescently labeled PCR products. Data were analyzed as combined carriage of an *FLG*-null allele; that is, children carrying 1 or more of the 6 genetic variations were considered to have an *FLG* loss-of-function mutation.^27^ In cases with incomplete *FLG* data, the presence of 1 *FLG* mutation defined that case as a carrier; participants with incomplete genotyping data in whom all alleles successfully tested were wild-type were excluded from further analysis because it was not possible to determine their *FLG* genotype status.^27^

### Statistical analysis

Allergen levels (expressed in micrograms per gram) underwent natural log transformation. The effects of *FLG* loss-of-function mutations and allergen exposure on allergen-specific sensitization at each age were investigated by using logistic regression. We first analyzed the associations between sensitization and allergen exposure in each *FLG* genotype group.

We then modeled the effect of the interaction between exposure and *FLG*, controlling for the main effects. Longitudinal analyses were performed by using generalized estimating equations (GEE). Population-averaged GEE models were developed to investigate whether the effect of allergen exposure, *FLG* loss-of-function mutation, and their interactions on the development of sensitization changed over time. Coefficients represent the increased/decreased odds of sensitization per log-unit increase in allergen exposure.

We also investigated the effect of cat and dog ownership in early life on sensitization. We adjusted all models with confounding variables, including sex, socioeconomic status, and breast-feeding.

We tested the assumption of a linear relationship between allergen exposure and sensitization by conducting likelihood ratio tests to compare the fit of nested models with inclusion and exclusion of a quadratic term for each exposure at all time points. Furthermore, link tests were carried out to check for model misspecification of the dependent variable when only a linear term for exposure was included.^35^

Given a smaller sample size at age 1 year, we assessed the sensitivity of our findings from longitudinal analyses with the exclusion of data at age 1 year. All analyses were conducted in Stata 15 software (StataCorp, La Jolla, Calif).

## Results

The flow of children through the study is summarized in Fig E1 in this article’s Online Repository at www.jacionline.org. Complete *FLG* genotyping was available for 712 (76.3%) of 933 white participants, of whom 131 did not have dust samples. We analyzed data from 581 children, of whom 51 (8.8%) had *FLG* loss-of-function mutations; 276 had complete sensitization data from age 3 to 16 years. Excluded participants were more likely to be male and have paternal asthma, but there were no differences in other risk factors and exposures, including *FLG* genotype and pet ownership (see Table E1 in this article’s Online Repository at www.jacionline.org).

Table E2 in this article’s Online Repository at www.jacionline.org shows the prevalence of sensitization from age 1 to 16 years in the whole population and stratified by *FLG* genotype. For all allergens, children with *FLG* mutations had significantly greater point prevalence of sensitization in preschool (age 1-5 years) and for cat and HDM by mid–school age (age 11 years), but there were no differences in sensitization between genotype groups in adolescence (age 16 years, see Fig E2 in this article’s Online Repository at www.jacionline.org).

### Early-life allergen exposure, FLG genotype, and allergen-specific sensitization

#### Cat

**Fig 1:**
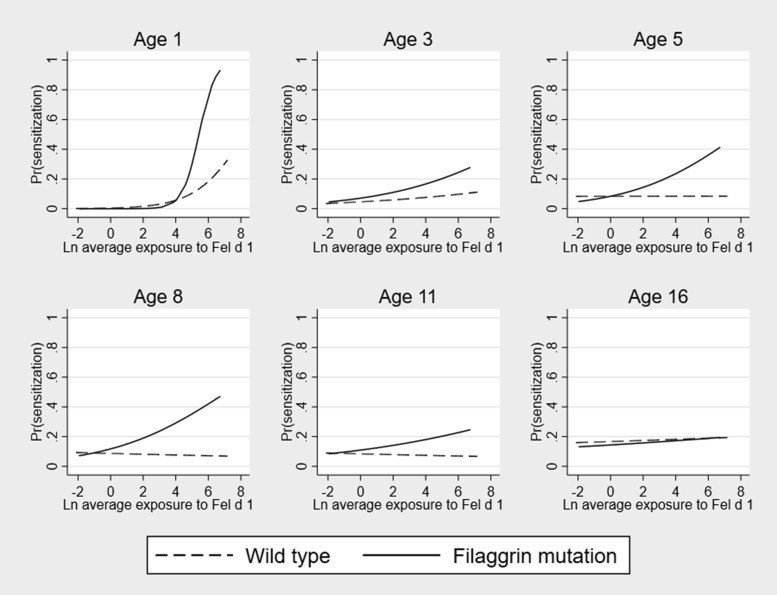
Effect of exposure to cat allergen Fel d 1 on the predicted probability of cat-specific sensitization among children with and without *FLG* loss-of-function mutations: ages 1, 3, 5, 8, 11, and 16 years. *Ln*, Natural log.

Cross-sectional analyses in each *FLG* genotype group suggested that the effect of early-life Fel d 1 exposure on cat sensitization differed between children with and without *FLG* mutations (Fig 1). Among children with mutations, an increase in exposure significantly increased the risk of sensitization at ages 1, 3, 5, and 8 years; this association was no longer significant thereafter (see Table E3 in this article’s Online Repository at www.jacionline.org). Among children with a wild-type genotype, there was a significant association between Fel d 1 exposure and cat sensitization at age 1 year, with no significant association at later ages. The effect of early-life exposure on sensitization diminished over time in both genotype groups. In longitudinal adjusted GEE models, Fel d 1 exposure significantly increased the risk of sensitization among children with *FLG* mutations (odds ratio [OR], 1.26; 95% CI, 1.04-1.52; *P* = .017), with no significant effect of exposure in those without mutations (OR, 0.94; 95% CI, 0.84-1.06; *P* = .34). Similar results were obtained in 230 children with complete data from age 3 to 16 years (see Table E3).

These observations suggesting an interaction between *FLG* genotype and Fel d 1 exposure were formally tested in models that included an interaction term controlling for main effects (Table I). The effect of early-life exposure on cat sensitization was significantly greater in the *FLG* mutation group at the ages of 5 years (OR, 1.99; 95% CI, 1.05-3.79; *P* = .035) and 8 years (OR, 1.59; 95% CI, 1.07-2.37; *P* = .02). In the longitudinal GEE model we observed a significant interaction between *FLG* genotype and Fel d 1 exposure in that the effect of early-life exposure on development of cat sensitization from infancy to age 16 years was significantly greater among children with *FLG* mutations compared with those without (OR, 1.36; 95% CI, 1.02-1.80; *P* = .035; Table I). The interaction effect remained robust to sensitivity testing among participants with complete data.

**Table I:** Multivariable analysis indicating effect of the interaction between *FLG* loss-of-function mutations and Fel d 1, Der p 1, and Can f 1 exposure on the risk of cat, mite, and dog allergen-specific sensitization

Age	Odds ratio	95% CI	*P* value
Interaction: *FLG* loss-off-function * Fel d 1 exposure, cat sensitization			
1 y	12.57	0.00-489810.02	.64
3 y	1.48	0.81-2.73	.20
5 y	1.99	1.05-3.79	.035
8 y	1.59	1.07-2.37	.021
11 y	1.34	0.85-2.12	.20
16 y	1.07	0.72-1.61	.73
GEE: age 1-16 y	1.36	1.02-1.80	.035
GEE: complete age 3-16 y	1.76	1.09-2.83	.021
Interaction: *FLG* loss-off-function * Der p 1 exposure, dust mite sensitization			
1 y	0.00	0.00- .	.99
3 y	1.00	0.60-1.65	.99
5 y	1.25	0.78-2.00	.36
8 y	0.97	0.64-1.46	.87
11 y	1.23	0.74-2.04	.42
16 y	0.93	0.54-1.60	.79
GEE: age 1-16 y	1.09	0.78-1.51	.62
GEE: complete age 3-16 y	0.91	0.53-1.56	.72
Interaction: *FLG* loss-off-function * Can f 1 exposure, dog sensitization			
1 y	0.55	0.15-2.05	.37
3 y	0.79	0.42-1.49	.47
5 y	0.94	0.51-1.70	.83
8 y	0.75	0.24-2.41	.63
11 y	0.45	0.06-3.34	.43
16 y	0.77	0.32-1.88	.57
GEE: age 1-16 y	0.76	0.47-1.22	.26
GEE: complete age 3-16 y	1.08	0.50-2.36	.84

*FLG* genotype*,* allergen exposure, sex, breast-feeding, and socioeconomic status are included as covariates.

#### HDM

**Fig 2:**
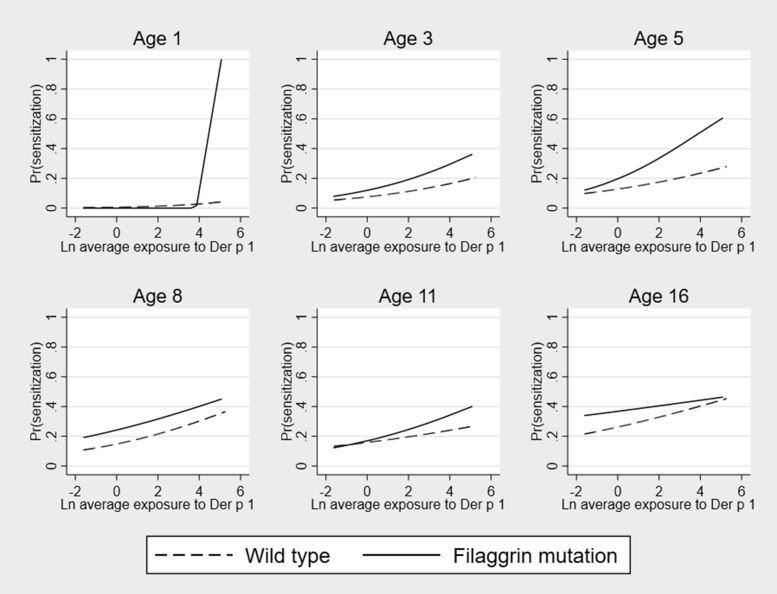
Effect of exposure to mite allergen Der p 1 on the predicted probability of mite-specific sensitization among children with and without *FLG* loss-of-function mutations: ages 1, 3, 5, 8, 11, and 16 years. *Ln*, Natural log.

Analyses in each *FLG* genotype group suggested a broad pattern similar to that observed for cat allergen (Fig 2). At age 1 year, the effect of Der p 1 exposure was markedly greater in children with *FLG* mutations (OR, 6.66; 95% CI, 1.15-38.58; *P* = .03); from age 3 years onward, ORs for the effect of Der p 1 exposure were numerically higher among children with *FLG* mutations, but this did not reach statistical significance (see Table E3). In longitudinal models we observed nonsignificant trends for the increase in risk of sensitization with increasing Der p 1 exposure in both genotype groups (OR, 1.11 [95% CI, 0.99-1.25; *P* = .06] and 1.31 [95% CI, 0.96-1.80; *P* = .09], wild-type and *FLG* mutations, respectively; see Table E3). Although the increase in risk per increase in unit of Der p 1 exposure was consistently greater among children with *FLG* mutations, except at age 16 years (see Table E3), in multivariable models the interaction between *FLG* and exposure did not reach statistical significance (Table I).

#### Dog

**Fig 3:**
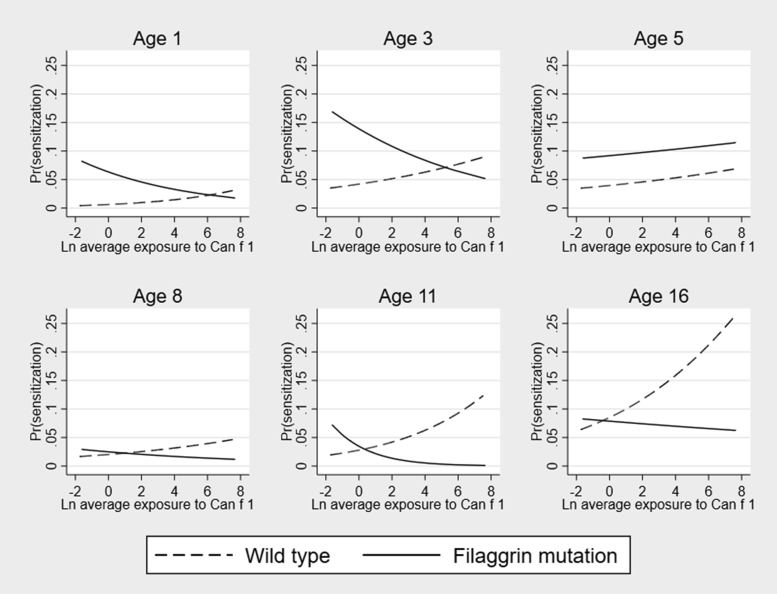
Effect of exposure to dog allergen Can f 1 on the predicted probability of dog-specific sensitization among children with and without *FLG* loss-of-function mutations: age 1, 3, 5, 8, 11, and 16 years. *Ln*, Natural log.

The relationship between Can f 1 exposure and dog sensitization differed from that observed for cat and HDM (Fig 3). Analyses in each genotype group showed that Can f 1 exposure in children without *FLG* mutations increased the risk of sensitization, with the effect being significant at age 16 years (OR, 1.26; 95% CI, 1.06-1.50; *P* = .001) and no significant effect of exposure among children with *FLG* mutations (see Table E3). In GEE models the effect of Can f 1 exposure differed between genotype groups, with a significant increase in the risk of sensitization among children without *FLG* mutations (OR, 1.20; 95% CI, 1.06-1.37; *P* = .004) but not among those with *FLG* mutations. The formal interaction analyses showed that the effect of Can f 1 exposure on dog sensitization was consistently lower at all ages in children with *FLG* mutations, but the interaction between *FLG* and dog allergen exposure did not reach significance (Table I).

For all allergens and models, a quadratic term did not improve the explanatory power of the relationship between exposure and sensitization (results are available on request).

### Pet ownership in the first year of life, *FLG* genotype, and sensitization during childhood

Fig E3, *A*, in this article’s Online Repository at www.jacionline.org shows the proportions of cat-sensitized children by *FLG* genotype and cat ownership in the first year of life. From infancy to age 11 years, children with *FLG* mutations and a cat at home had the greatest risk of sensitization; the probability of sensitization converged to a similar level by age 16 years in all 4 groups (see Fig E4 in this article’s Online Repository at www.jacionline.org). Adjusted cross-sectional analyses (see Table E4 in this article’s Online Repository at www.jacionline.org) consistently showed that from age 3 to 8 years, children with *FLG* mutations and a cat at home had the greatest risk of cat sensitization (approximately 4-fold greater risk compared with those without mutations and no cat). Longitudinal analyses demonstrated that children with *FLG* mutations and a cat at home had the greatest probability of cat sensitization during childhood, which was significantly greater compared with children with no cat and wild-type *FLG* (OR, 3.02; 95% CI, 1.26-7.21; *P* = .013; see Table E4). The results of the adjusted longitudinal model are presented in Table E5 in this article’s Online Repository at www.jacionline.org.

These relationships were different for dog ownership. Children with *FLG* mutations and a dog at home had the lowest point prevalence of dog sensitization at all ages apart from 5 years (see Fig E3, *B*). In this group there were no dog-sensitized subjects at 5 follow-ups, rendering the results of cross-sectional analyses uncertain (see Table E6 in this article’s Online Repository at www.jacionline.org). Models indicated that dog ownership among children with *FLG* mutations was protective (see Fig E5 in this article’s Online Repository at www.jacionline.org), but formal statistical significance of the interaction in GEE models was not achieved (OR, 0.06; 95% CI, 0.00-1.72; *P* = .10; see Table E7 in this article’s Online Repository at www.jacionline.org).

**Fig 4:**
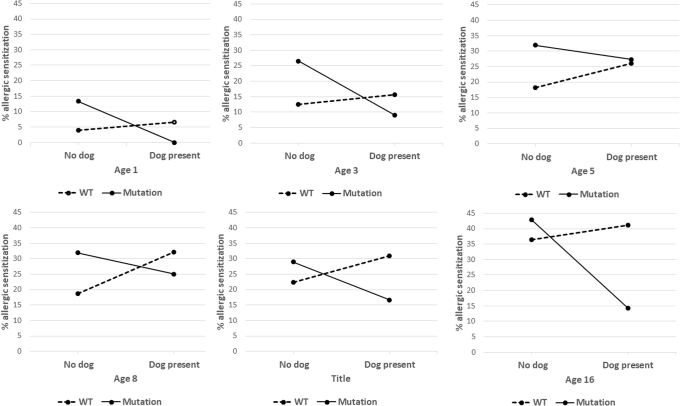
Proportion of children with allergic sensitization (assessed by using skin prick tests]) by *FLG* genotype and dog ownership in early childhood from age 1 to age 16 years. *WT*, Wild-type.

To investigate whether dog ownership can offer protection that is not allergen specific, we proceeded to analyze sensitization to any allergen. Fig E6 in this article’s Online Repository at www.jacionline.org shows the proportions of sensitized children according to *FLG* genotype and dog ownership. A consistent finding was decreased risk of sensitization among dog owners with *FLG* mutations, with the opposite effect in those without mutations (Fig 4). In the longitudinal GEE model there was a significant interaction between *FLG* genotype and dog ownership in that the risk of sensitization was markedly and significantly lower among children with *FLG* mutations who had a dog in the home in infancy (OR, 0.16; 95% CI, 0.03-0.86; *P* = .03; Table II). There was no effect of cat ownership on sensitization to any allergen and no interaction with *FLG* (see Table E8 in this article’s Online Repository at www.jacionline.org).

**Table II:** Adjusted GEE analyses showing the interaction effect of dog ownership and *FLG* loss-of-function mutation on the development of allergic sensitization from age 3 to 16 years

	Allergic sensitization (n = 483)	
	OR (95% CI)	*P* value
Dog present (first year of life)	1.56 (0.98-2.47)	.061
*FLG* loss-of-function mutation	2.27 (1.20-4.32)	.012
Dog present * *FLG* mutations	0.16 (0.03-0.86)	.032
Age	1.08 (1.06-1.10)	.000
Male sex	1.81 (1.24-2.63)	.002
Breast-feeding	1.00 (0.65-1.54)	.994
Socioeconomic status (managerial level)	1.23 (0.83-1.83)	.306

Dog ownership refers to the presence of a dog in the first year of life. Sensitization is defined as at least 1 positive test result to *Dermatophagoides pteronyssinus*, cat, dog, grass pollen, molds, milk, and egg (ages 1-5 years) and birch and peanut (ages 8-16 years; ie, a total of 9 allergens).

## Discussion

In this population-based birth cohort children with *FLG* mutations were more likely to be sensitized to inhalant allergens from infancy to school age, but there were no differences in sensitization between those with and without *FLG* mutations in adolescence. Longitudinal sensitization trajectories differed between children exposed to an allergen or allergens in the first year of life compared with those not exposed and between genotype groups. In general, the effect of cat and mite allergen exposure on allergen-specific sensitization was greater among children with *FLG* loss-of-function mutations compared with those without. We have shown a significant interaction between early-life cat allergen exposure and the *FLG* genotype on the development of cat sensitization during childhood, and the effect of early-life exposure was significantly greater among children with *FLG* mutations (approximately 36% increase in risk per log-unit increase in Fel d 1 in children with *FLG* mutations compared with those without). *FLG* mutations significantly increased the effect of early-life Der p 1 exposure on mite sensitization at age 1 year, but this modifying effect was gradually reduced over time. Markedly different patterns of the relationship between *FLG* genotype and exposure to dog on sensitization were observed in that the risk of dog sensitization appeared lower, and the risk of sensitization to any allergen was significantly lower among children with *FLG* mutations who were exposed to a dog in infancy (on average, >5-fold reduction in the risk of sensitization during childhood).

### Limitations and strengths

The main limitation of our study is the lack of a replication population. However, there are very few birth cohorts that have objective measures of exposure to multiple allergens in early life and that have assessed sensitization on multiple occasions from early childhood to adolescence, both of which are key to interpreting our findings. Also, we were unable to include all cohort participants because of the availability of early-life dust samples and *FLG* genotyping.

The 6 *FLG* mutations we assessed have been consistently associated with eczema in white populations^36^; however, because some of these mutations are not found in nonwhite subjects, all nonwhite participants were excluded from our analyses, and the results are not applicable to other ethnicities. Our definition of loss-of-function mutations within the *FLG* gene included carrying 1 or more of the 6 genetic variations. As a result, among participants with incomplete genotyping data in whom all alleles successfully tested were wild-type, it was not possible to determine their *FLG* status, and these subjects were excluded from further analysis, with a consequent reduction in sample size. We repeated our analyses using *FLG* variants 2282del4 and R501X only, and the results were entirely consistent with findings when FLG status was defined by using all 6 mutations, with no material change in any of the reported significant interactions (data available on request).

Another limitation is the smaller sample size at age 1 year and in some of the subgroups (eg, sensitized dog owners with *FLG* mutations), and our findings need to be interpreted with caution. We could not address the question about the relative importance of exposure in infancy compared with that in later childhood.

The strengths of this study include comprehensive measurements of early-life allergen exposure and objective evaluation of sensitization from infancy to adolescence. Sensitization was assessed at 6 time points, which allowed analysis of the effect of allergen exposure and genotype over time. We used data on both pet allergen exposure and pet ownership, and similar findings in these 2 measures of exposure strengthen our findings.

### Interpretation

To our knowledge, this is the first study to investigate the relationship between objectively measured exposure to inhalant allergens and *FLG* mutations with longitudinal trajectories of allergic sensitization. We have previously shown that *FLG* loss-of-function mutations modify the effect of environmental peanut exposure on the development of peanut sensitization and allergy.^27^ Our current study extends this to inhalant allergens and suggests that the transcutaneous route through an impaired skin barrier might be important for sensitization. However, although there is currently no consensus about the presence of FLG in respiratory tissues,^37^ we cannot exclude the possibility that the effects observed in this study are mediated through the inhaled route and exposure in the nose because FLG might be expressed in human nasal mucosa.^38^

Two birth cohorts in the United Kingdom and Denmark have shown a significant interaction between *FLG* loss-of-function mutations and early-life cat ownership on the development of infantile atopic dermatitis (AD).^26^ In the birth cohort from The Netherlands, early-life cat ownership enhanced the effect of *FLG* mutations on AD at ages 4 and 8 years but, similar to our results, not on sensitization to any allergen.^39^ A significant association has been reported between the severity of AD and cat sensitization in *FLG*-related AD but not in non–*FLG*-related AD,^40^ and one mechanism by which cat exposure could drive the development of AD is by enhancing cat-specific sensitization facilitated though an impaired skin barrier. In the current study in children with wild-type *FLG*, Fel d 1 exposure increased the risk of cat sensitization at age 1 year, but this association diminished as children got older. In contrast, in children with *FLG* mutations, the increased risk of cat sensitization related to high allergen exposure in infancy persisted over time, with different trajectories of sensitization during childhood in children with different genotypes in relation to the same environmental exposure. By age 16 years, the point prevalence of cat-specific sensitization was the same in children with and without a cat in both genotype groups. This might be in part due to exposure to cat allergen outside the home because previous studies have shown that cat allergen is transported on clothing and can be measured at high levels in homes without cats and public places.^33^^,^^41^

*FLG* mutations significantly increased the effect of Der p 1 exposure on mite sensitization at age 1 year. After this, the interaction between *FLG* and exposure decreased considerably. It has been shown that early sensitization (including that to mite and cat) is crucially important for the development of asthma,^42^, ^43^, ^44^, ^45^ and our finding that the interactions between *FLG* and cat and mite exposure in relation to specific sensitizations are stronger in early life might be one of the mechanisms by which *FLG* loss-of-function mutations increase the risk of asthma.^24^

Mite allergens have proteolytic activity,^46^ which can disrupt the skin barrier by cleaving tight junction proteins.^47^ Thus mite allergens might disrupt the skin barrier without the increased susceptibility of *FLG* loss-of-function mutations. In support of this, in BALB/c mice recombinant Der p 1 was able to induce eczema without skin stripping or addition of an adjuvant.^48^ However, it is unclear whether the magnitude of exposure in the animal model would resemble skin exposure in infants in real life, and there are no definitive studies to confirm this.

The effect of exposure to dogs differed from that observed for cats and mites in that dog ownership (and high exposure to dog allergen) was protective among children with *FLG* loss-of-function mutations and that this protective effect extended to sensitization to any allergen. Dog ownership can offer protection through the increase in microbial exposure,^1^^,^^49^ and our finding of a significantly stronger protective effect among subjects with *FLG* mutations can be explained by comparatively greater personal exposure to microbial products consequent to the impaired skin barrier. The fact that significant effects of Fel d 1 exposure and cat ownership were confined to cat-specific sensitization (but not sensitization to other allergens) suggests that the observed effects are related to allergen exposure. Taken together, our data support the notion that differences in the effects of cat and dog ownership might be a consequence of a cat being a marker of high allergen exposure, whereas the protective effect of dog exposure can be mediated through changes in the skin microbiome.^50^

The findings of the current study confirm our previous observation of the changing nature of the association between early-life exposures and sensitization with time and the crucial importance of longitudinal analyses.^15^ Furthermore, they raise fundamental questions about the current approach to replication in genetic and gene-environment studies because the timing of the assessment of outcomes can critically affect the results of different studies investigating genes, the environment, and their interactions. Where there are inconsistencies, we should move from direct replication toward triangulation (ie, integration of evidence from several approaches with differing and unrelated sources of bias) to improve causal inference.^51^

In conclusion, *FLG* loss-of-function mutations modify the relationship between early-life allergen exposure and sensitization, but effects differ at different ages and between different allergens. Children with *FLG* mutations might benefit from mite and cat avoidance but can gain from having a dog in early life, but this will have to be confirmed in prospective studies.

**Clinical implications**

Children with *FLG* mutations might benefit from mite and cat avoidance but might gain from having a dog in early life.

## Supplementary data

Online Repository text
