# Supplementary material for: Early-life inhalant allergen exposure, filaggrin genotype, and the development of sensitization from infancy to adolescence
Source: J Allergy Clin Immunol. 2020 Mar;145(3):993–1001. doi: 10.1016/j.jaci.2019.08.041 (PMC7057264; doi:10.1016/j.jaci.2019.08.041)
Supplement: Online Repository text [file mmc1.docx]

**Early-life inhalant allergen exposure, filaggrin genotype and the development of sensitization from infancy to adolescence**

Angela Simpson MD PhD^1*^, Helen A Brough MD PhD^2,3,4*^, Sadia Haider PhD^5,6*^, Danielle Belgrave PhD^7^, Clare S Murray MD^1^, Adnan Custovic MD PhD FAAAI^5,6^

^1^Division of Infection, Immunity and Respiratory Medicine, Faculty of Biology, Medicine and Health, Manchester Academic Health Sciences Centre, University of Manchester and University Hospital of South Manchester NHS Foundation Trust, Manchester, UK

^2^ Children's Allergy Service, Evelina London, Guys and St Thomas' NHS Trust, London, UK

^3^ Paediatric Allergy Group, Department of Women and Children's Heath,

School of Life Course Sciences, London, UK

^4^ Paediatric Allergy Group, School of Immunology & Microbial Sciences,

King's College London, London, UK

^5^Section of Paediatrics, Imperial College London, UK

^6^National Heart and Lung Institute, Imperial College London, UK

^7^Microsoft Research Cambridge

*Equal contribution, joint first authors

**Online Repository**

**METHODS**

***Screening & Recruitment***

All pregnant women were screened for eligibility at antenatal visits (8^th^-10^th^ week of pregnancy). Of the 1499 couples who met the inclusion criteria (<10 weeks of pregnancy, maternal age >18 years), 288 declined to take part and 27 were lost to follow-up between recruitment and birth of a child. A total of 1184 participants had some evaluable data.

***Follow-up***

Children were followed prospectively, and attended review clinics at ages 1, 3, 5, 8, 11, and 16 years. At age 1 year, only children with either both atopic parents, or no atopic parents who lived in homes without a pet were invited to attend clinical follow up. At all other time points for all other measures all children were invited to participate.

*Allergic sensitization*. Sensitization was ascertained by skin prick tests (SPT) at all ages for 7 allergens (Dermatophagoides pteronyssinus, cat, dog, grass pollen, molds, milk, and egg [Bayer, Elkahrt, Ind, US]). From age 8 years, SPTs were additionally performed for tree pollen (birch) and peanut (total of 9 allergens tests). We defined sensitization as a mean wheal diameter 3 mm larger than that elicited by the negative control to at least 1 of the allergens tested.

***Quantitation of environmental Der p 1, Fel d 1 and Can f 1 exposure in household dust***

Dust samples were collected using a Dust Sampler (Medivac plc, Wilmslow, UK) with air-flow rate 45 l/sec, through a 355 μm diameter mesh screen on to a 5 μm vinyl filter (Plastok Associates Ltd, Wiral, UK). The filters were supported in a plastic dust trap located behind the cleaner attachment (nozzle). This method enabled collection of fine dust samples. Each sample was transferred into a pre-weighed petri dish, weighed, coded and stored at 4^o^C.

One hundred mg of fine dust was extracted with 2 ml borate-buffered saline with 0.1% Tween 20 (BBS-T), pH 8.0. For samples less than 50 mg 1 ml BBS-T was added. Samples between 50 mg and 100 mg were extracted in the proportional amount of BBS-T (1:20). The dust was re-suspended using a vortex mixer (Vortex-Genie, Fisher Scientific, USA). Samples were then mixed end over end on an orbital rotator (Rotator SB-1, Stuart Scientific, UK) for 2 hours at room temperature before being centrifuged for 20 minutes at 2500 revolutions per minute (RPM) at 4^o^C (BR 401 Refrigerated Centrifuge, Denley, UK). Supernatants were removed with a Pasteur pipette, and the dust pellets discarded. Extracts (supernatants) were stored at -20^o^C for future analysis of allergen content.

Quantitative assessment of allergen exposure was performed using a monoclonal antibody (mAb) based assay.(1-3) Freeze-dried mAbs were resuspended in 1-ml distilled water to a concentration of 10 mg/ml. Immulon II flat bottom ELISA plates were coated with 1 μl/well of 6F9 mAb (Fel d 1) and 5H8 (Der p 1) in 0.05 M carbonate-bicarbonate buffer pH 9.6 overnight at 4^o^C. Plates were washed twice with PBS-T pH 7.4 and incubated for 1 hour with 1% BSA PBS-T. After 2 further washes, 0.1 ml of diluted allergen samples was added. Standards for Fel d 1 (UVA 91/01) and Der p 1 (UVA 93/02) were diluted across the plate in duplicate to establish the control curves. The plates were incubated for 1 hour at room temperature, and then washed 5 times and incubated for 1 hour with 0.1 ml/well of biotinylated 3E4 (Fel d 1) or 5H8 (Der p 1) mAbs. 100μl 1/1000 dilution of Streptavidin-peroxidase was added after a further 5 washes, and the plates were incubated for 30 minutes. Plates were washed a final 5 times, and the assay developed by adding 0.1 ml 1 mM 2.2'-azino-bis (3-ethylbenzthiazoline-6-sulphonic acid) in 0.07 M citrate phosphate buffer pH 4.2 containing 0.03% H_2_O_2_ added immediately before.

UVA 91/01 standard for Fel d 1 contained 2 Units Fel d 1/ml (relative to CBER Cat E5 standard containing 9.7 Units/ml; 1Unit = 4μg protein). The standard used to establish the control curve for Der p 1 assay (UVA 93/02) was considered to contain 2500 ng Der p 1/mL (relative to WHO/IUIS *Dermatophagoides pteronyssinus* standard NIBSC 82/518 which has been estimated to contain 12.5 µg Der p 1 per ampoule). Doubling dilutions of this standard extract were used to construct a control curve with each individual assay; the dilutions used were 250 - 0.5 ng/mL. Can f 1 assay was quantified by using dog allergen standard (UVA 94/02; 10,000 IU Can f 1/mL) sub-standardized against WHO/IUIS International Reference Preparation of dog hair and dander (NIBSC 84/685), which contains 100,000 IU/mL Can f 1 (1 IU = 1 ng Can f 1 protein).

Allergen levels are reported in micrograms of allergen per gram of fine dust. Concentrations ≤0.200 µg/g were regarded as below the detection limit of the assay and were assigned a value of 0.1 µg/g for the purposes of analysis. Allergen levels were log-transformed (Ln(x)) in order to make the variable normally distributed.

**RESULTS**

**Figure E1:** Participant flow indicating children included in the analysis


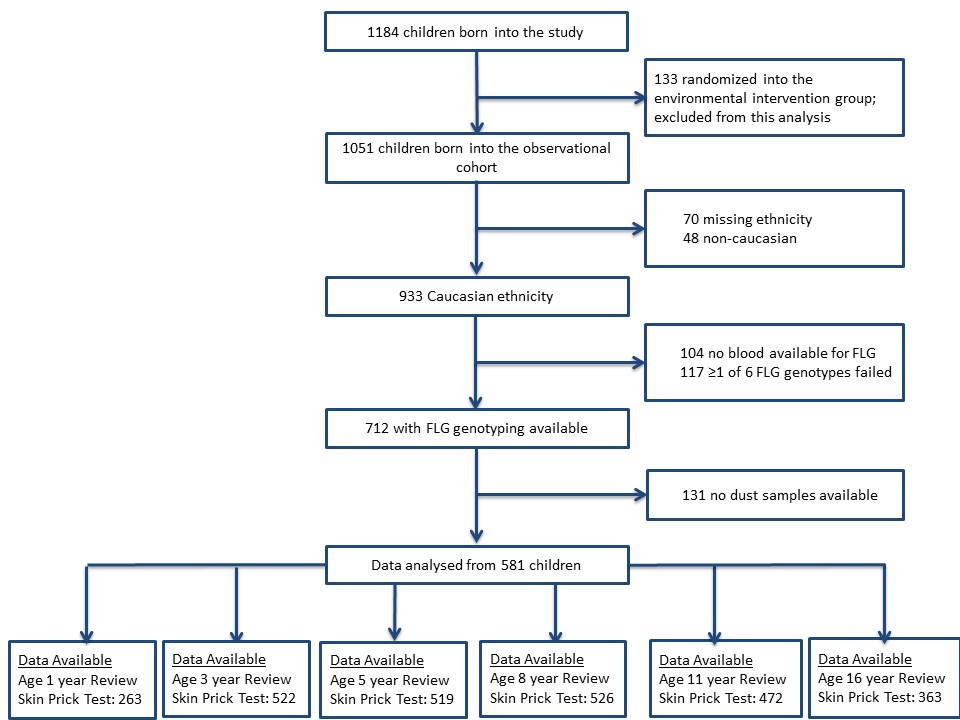


**Figure E2:** Profile plot showing the cross-sectional trends in the probability of cat (A), mite (B) and dog sensitisation (C) among children with and without *FLG* mutations from age 1 to 16 years.

**A**


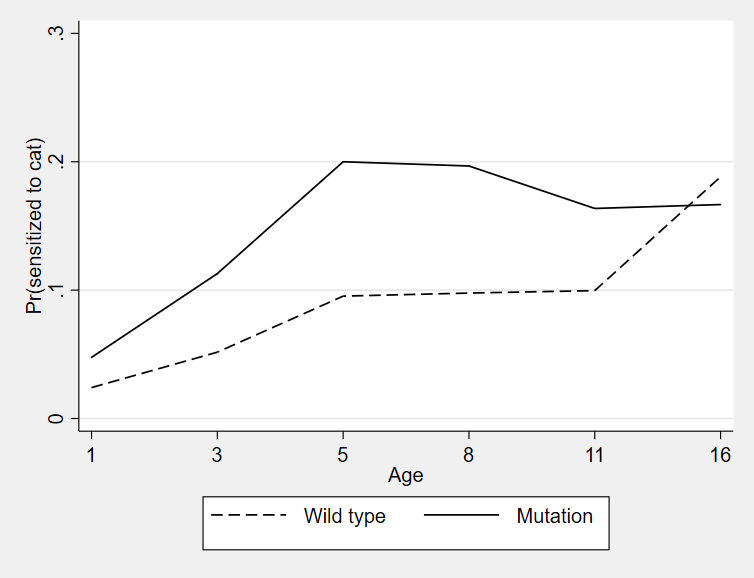


**B**


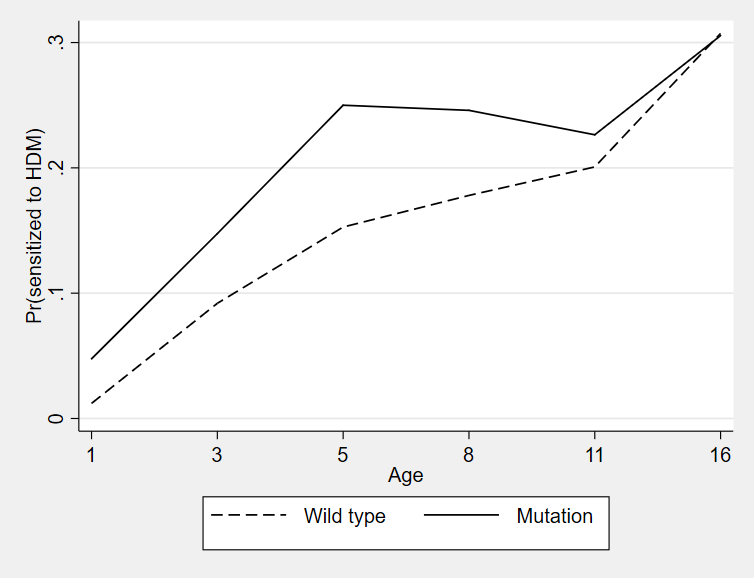


**C**


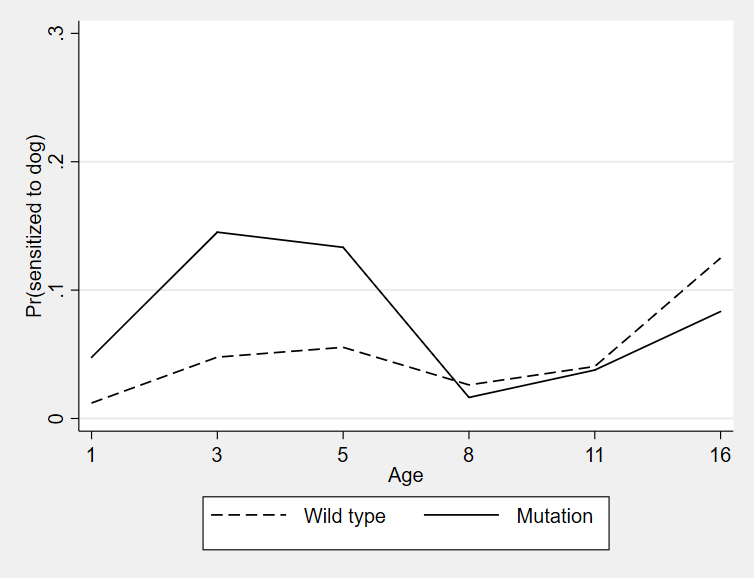


**Figure E3:** Profile plot showing the effect of cat (A) and dog (B) ownership in the first year of life on the probability of allergen-specific sensitization in children with and without *FLG* loss-of-function mutations.

**A**


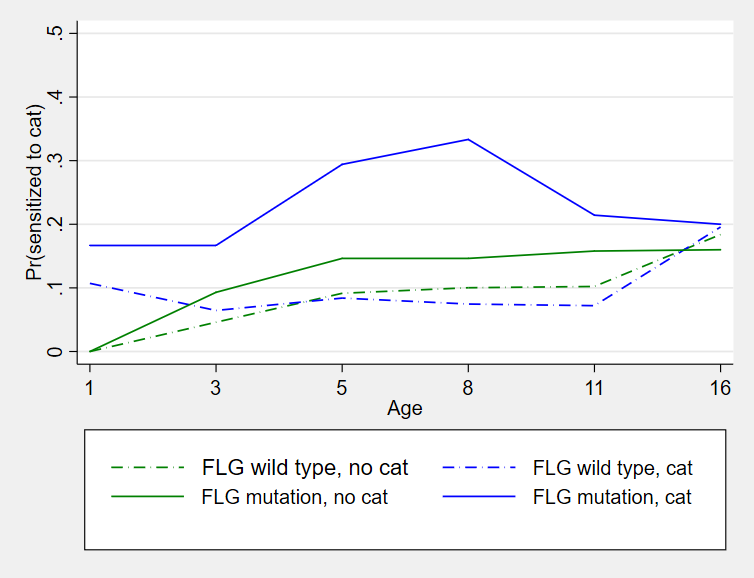


**B**


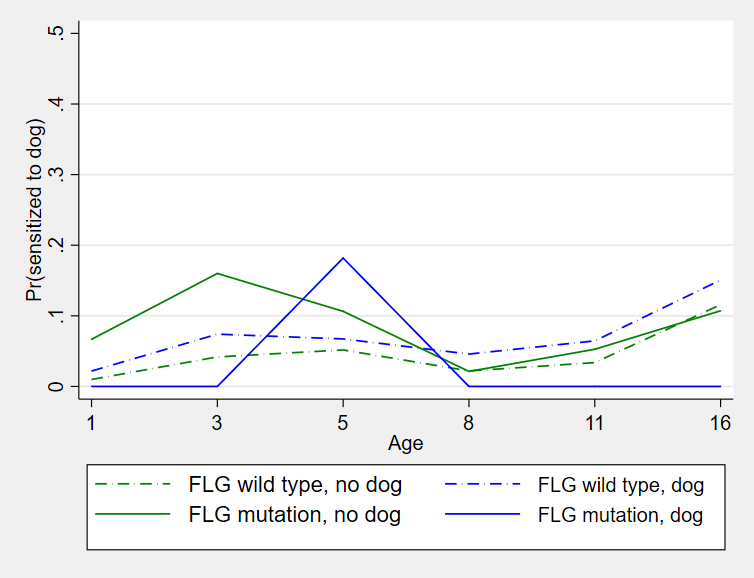


**Figure E4.** Percentage of children with cat sensitization (assessed by SPT) by *FLG* genotype and cat ownership in early childhood from age 1 to age 16 years.


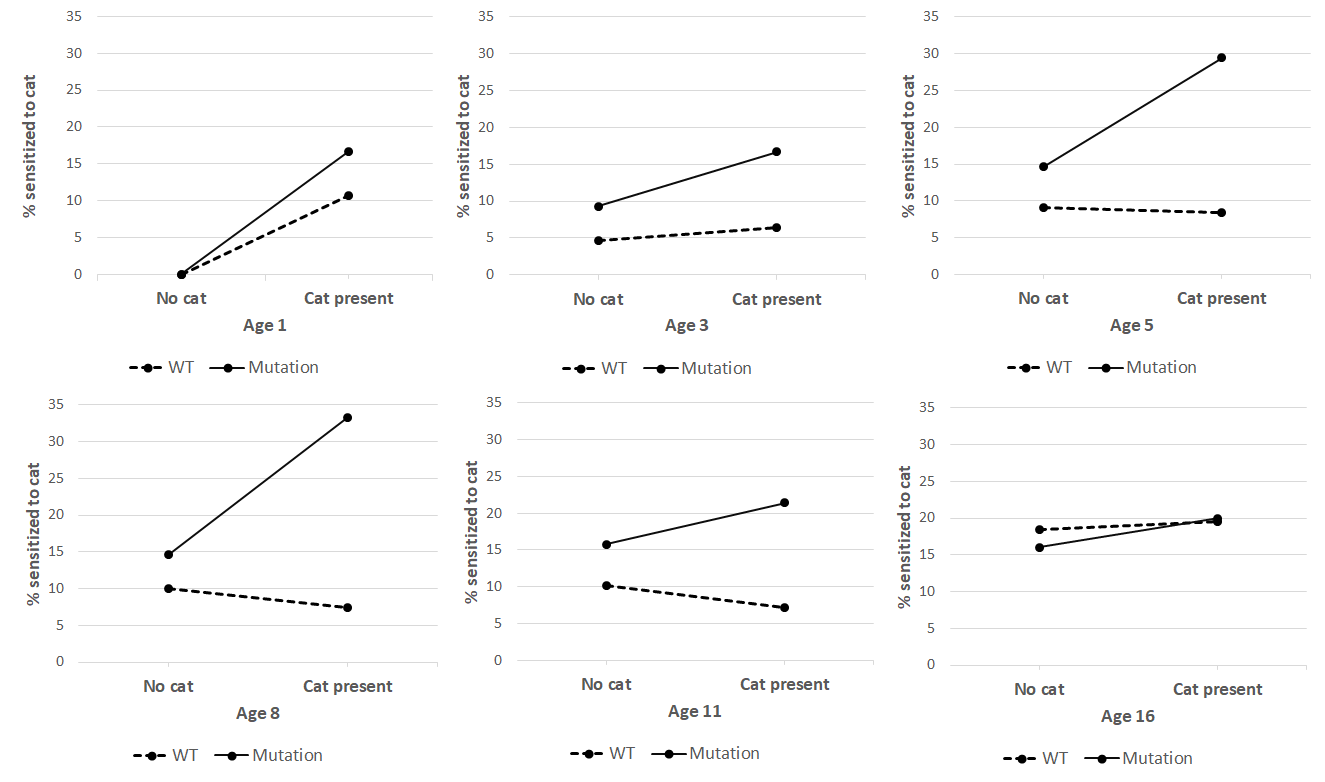


**Figure E5.** Percentage of children with dog sensitization (assessed by SPT) by *FLG* genotype and dog ownership in early childhood from age 1 to age 16 years.


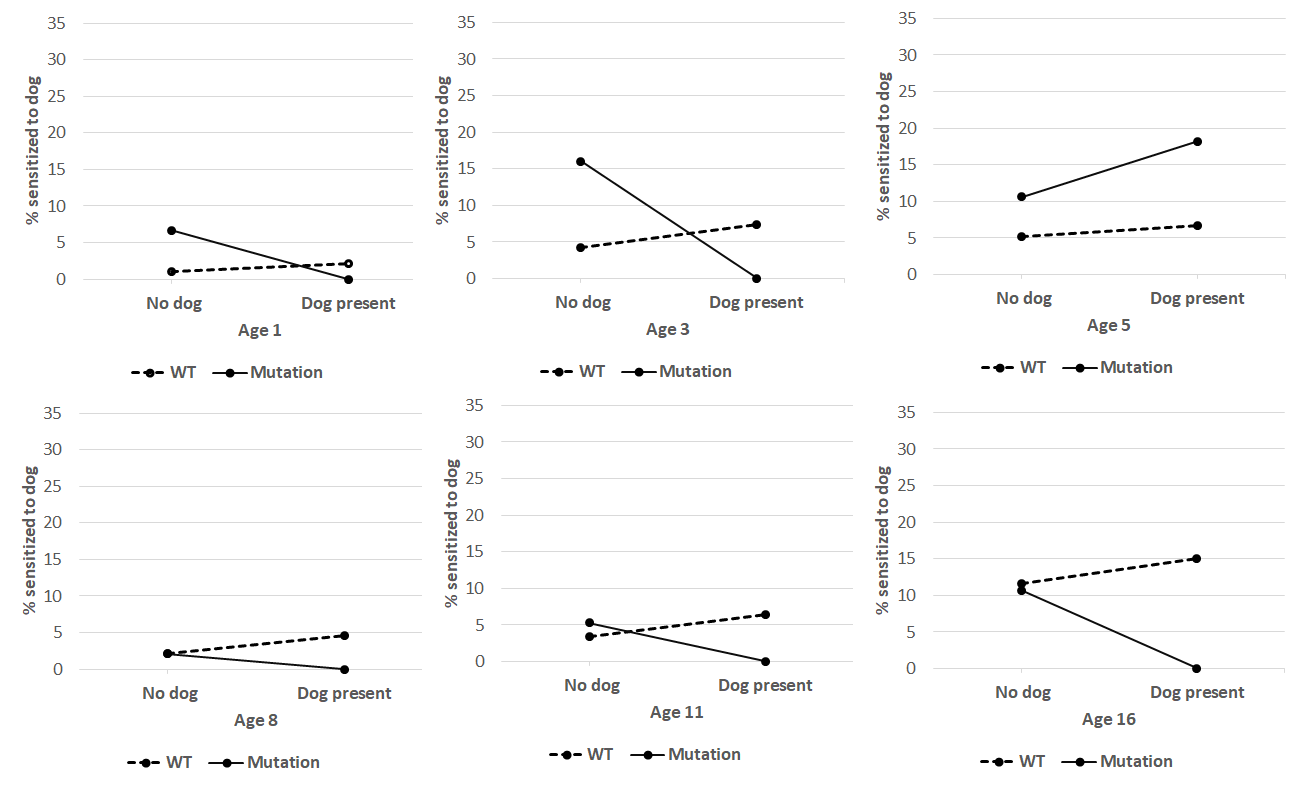


**Figure E6:** Profile plot showing the effect of dog ownership in the first year of life on the probability of allergic sensitization in children with and without *FLG* loss-of-function mutations.


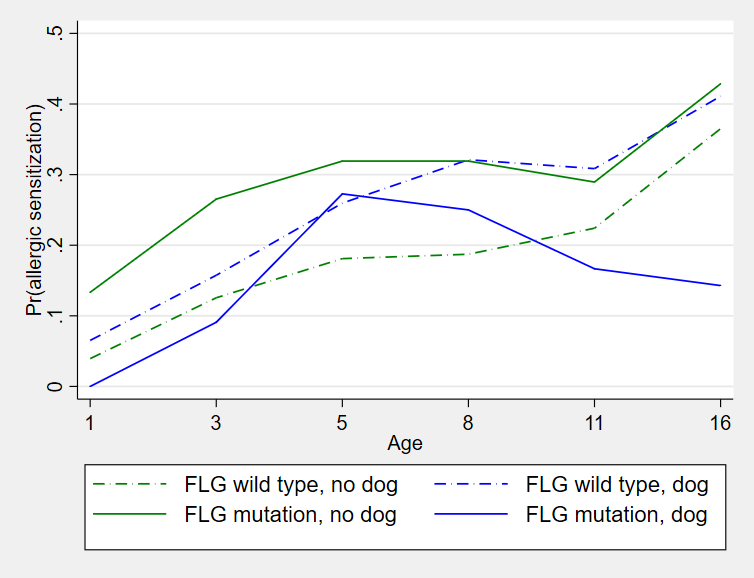


**Table E1:** Comparison of characteristics of children included and excluded from analysis

| **Characteristics** | **Whole cohort (n=1051)**  n (%) | **Included**  **(n=581)**  n (%) | **Excluded (n=470)**  n (%) | ***P*-value** |
| --- | --- | --- | --- | --- |
| **Filaggrin loss of function mutation** | 70 (9.2) | 51 (8.8) | 19 (10.6) | 0.458 |
| **Gender (Male)** | 580 (55.2) | 300 (51.6) | 280 (59.6) | 0.010 |
| **Maternal Asthma** | 199 (18.9) | 109 (18.8) | 90 (19.2) | 0.873 |
| **Paternal Asthma** | 126 (12.0) | 58 (10.0) | 68 (14.5) | 0.024 |
| **Maternal Atopy** | 549 (54.2) | 312 (53.8) | 237 (54.7) | 0.766 |
| **Paternal Atopy** | 583 (58.1) | 342 (59.0) | 241 (56.8) | 0.500 |
| **Cat ownership (first year of life)** | 240 (23.4) | 143 (24.6) | 97 (21.8) | 0.282 |
| **Dog ownership (first year of life)** | 201 (19.6) | 115 (19.8) | 89 (19.3) | 0.838 |
| **Breastfeeding Ever** | 682 (69.2) | 404 (70.5) | 278 (67.3) | 0.113 |
| **Day-care Ever** | 642 (67.3) | 391 (68.7) | 251 (65.2) | 0.399 |
| **Maternal smoking during pregnancy** | 108 (11.9) | 65 (11.7) | 43 (12.3) | 0.768 |
| **Any Smoke exposure during pregnancy** | 291 (32.1) | 177 (31.8) | 114 (32.7) | 0.781 |
| **Socio-economic status (Managerial)** | 370 (61.6) | 281 (62.3) | 89 (59.3) | 0.517 |
|  | **mean (sd)** | **mean (sd)** | **mean (sd)** |  |
| **Gestational Age** | 39.9 (1.62) | 39.9 (0.06) | 39.8 (0.08) | 0.199 |
| **Birth Weight (kg)** | 3.4 (0.51) | 3.4 (0.02) | 3.4 (0.02) | 0.804 |

**Table E2:** The prevalence of sensitisation to HDM, cat, dog and any allergen from age 1 to age 16 years in the whole population and stratified by *FLG* genotype.

|  | **Cat sensitisation** | | | | | |  |
| --- | --- | --- | --- | --- | --- | --- | --- |
|  | **Sensitised** | | **Sensitised + *FLG* wild type** | | **Sensitised + *FLG* LOF mutation** | |  |
| **Age** | **Frequency** | **%** | **Frequency** | **%** | **Frequency** | **%** | **P-value** |
| **1** | 7/270 | 2.59 | 6/249 | 2.41 | 1/21 | 4.76 | 0.436 |
| **3** | 35/604 | 5.79 | 28/542 | 5.17 | 7/62 | 11.29 | 0.076 |
| **5** | 67/637 | 10.52 | 55/577 | 9.53 | 12/60 | 20 | 0.024 |
| **8** | 68/634 | 10.73 | 56/573 | 9.77 | 12/61 | 19.67 | 0.027 |
| **11** | 61/577 | 10.57 | 52/522 | 9.96 | 9/55 | 16.36 | 0.163 |
| **16** | 83/445 | 18.65 | 77/409 | 18.83 | 6/36 | 16.67 | 1.000 |
|  | **Mite sensitisation** | | | | | |  |
|  | **Sensitised** | | **Sensitised + *FLG* wild type** | | **Sensitised + *FLG* LOF mutation** | | **P-value** |
| **Age** | **Frequency** | **%** | **Frequency** | **%** | **Frequency** | **%** |  |
| **1** | 4/270 | 1.48 | 3/249 | 1.2 | 1/21 | 4.76 | 0.278 |
| **3** | 59/605 | 9.75 | 50/544 | 9.19 | 9/61 | 14.75 | 0.172 |
| **5** | 103/636 | 16.19 | 88/576 | 15.28 | 15/60 | 25 | 0.064 |
| **8** | 117/634 | 18.45 | 102/573 | 17.8 | 15/61 | 24.59 | 0.223 |
| **11** | 116/571 | 20.32 | 104/518 | 20.08 | 12/53 | 22.64 | 0.720 |
| **16** | 137/446 | 30.72 | 126/410 | 30.73 | 11/36 | 30.56 | 1.000 |
|  | **Dog sensitisation** | | | | | |  |
|  | **Sensitised** | | **Sensitised + *FLG* wild type** | | **Sensitised + *FLG* LOF mutation** | | **P-value** |
| **Age** | **Frequency** | **%** | **Frequency** | **%** | **Frequency** | **%** |  |
| **1** | 4/270 | 1.48 | 3/249 | 1.2 | 1/21 | 4.76 | 0.278 |
| **3** | 35/606 | 5.78 | 26/544 | 4.78 | 9/62 | 14.52 | 0.006 |
| **5** | 40/637 | 6.28 | 32/577 | 5.55 | 8/60 | 13.33 | 0.043 |
| **8** | 16/634 | 2.52 | 15/573 | 2.62 | 1/61 | 1.64 | 1.000 |
| **11** | 23/571 | 4.03 | 21/518 | 4.05 | 2/53 | 3.77 | 1.000 |
| **16** | 54/444 | 12.16 | 51/408 | 12.05 | 3/36 | 8.33 | 0.601 |
|  | **Allergic sensitization to any allergen tested*** | | | | | |  |
|  | **Sensitised** | | **Sensitised + *FLG* wild type** | | **Sensitised + *FLG* LOF mutation** | |  |
| **Age** | **Frequency** | **%** | **Frequency** | **%** | **Frequency** | **%** | **P-value** |
| **1** | 13/270 | 4.81 | 11/249 | 4.42 | 2/21 | 9.52 | 0.268 |
| **3** | 88/604 | 14.57 | 73/543 | 13.44 | 15/61 | 24.59 | 0.033 |
| **5** | 134/637 | 21.04 | 115/577 | 19.93 | 19/60 | 31.67 | 0.045 |
| **8** | 143/634 | 22.56 | 125/573 | 21.82 | 18/61 | 29.51 | 0.197 |
| **11** | 140/572 | 24.48 | 126/519 | 24.28 | 14/53 | 26.42 | 0.738 |
| **16** | 164/442 | 37.10 | 151/406 | 37.19 | 13/36 | 36.11 | 1.000 |

*Sensitization was ascertained by skin prick tests (SPT) at all ages for 7 allergens (Dermatophagoides pteronyssinus, cat, dog, grass pollen, molds, milk, and egg [Bayer, Elkahrt, Ind, US]). From age 8 years, SPTs were additionally performed for tree pollen (birch) and peanut (total of 9 allergens tests). We defined sensitization as a mean wheal diameter 3 mm larger than that elicited by the negative control to at least 1 of the allergens tested.

**Table E3:** Multivariable regression analyses indicating the effect of Fel d 1, Der p 1, and Can f 1 exposure on the risk of cat, mite, and dog sensitization respectively. Analysis conducted in both *FLG* genotype groups at each age and across all time-points by Generalized Estimating Equations (GEE). Sex, breastfeeding, socio-economic status, and age included as covariates.

|  | **Filaggrin Wild Type** | | | | **Filaggrin Loss-of-Function Mutations** | | | |
| --- | --- | --- | --- | --- | --- | --- | --- | --- |
| ***Age*** | Odds Ratio | (95% CI) | p-value | n | Odds Ratio | (95% CI) | p-value | n |
| ***Fel d 1 exposure and cat sensitisation*** | | | | | | | |  |
| **1 year** | 1.98 | (1.19 - 3.28) | 0.008 | 183 | 2.23 | (1.09 - 4.57) | 0.029 | 16 |
| **3 years** | 1.12 | (0.92 - 1.36) | 0.273 | 368 | 1.39 | (1.05 - 1.86) | 0.023 | 39 |
| **5 years** | 0.90 | (0.74 - 1.09) | 0.293 | 366 | 1.35 | (1.04 - 1.74) | 0.025 | 33 |
| **8 years** | 0.92 | (0.77 - 1.09) | 0.313 | 372 | 1.39 | (1.10 - 1.77) | 0.007 | 36 |
| **11 years** | 0.89 | (0.72 - 1.09) | 0.254 | 329 | 1.23 | (0.88 - 1.71) | 0.222 | 29 |
| **16 years** | 0.95 | (0.82 - 1.10) | 0.495 | 271 | 1.04 | (0.75 - 1.46) | 0.808 | 23 |
| **GEE: age 1-16** | 0.94 | (0.84 - 1.06) | 0.338 | 401 | 1.26 | (1.04 - 1.52) | 0.017 | 40 |
| **GEE: complete age 3-16** | 0.89 | (0.76 - 1.05) | 0.172 | 216 | 1.42 | (1.09 - 1.86) | 0.010 | 14 |
| ***Der p 1 exposure and HDM sensitisation*** | | | | | | | | |
| **1 year** | 1.66 | (0.67 - 4.15) | 0.275 | 191 | 6.66 | (1.15 - 38.58) | 0.034 | 17 |
| **3 years** | 1.15 | (0.95 - 1.41) | 0.153 | 377 | 1.28 | (0.80 - 2.05) | 0.308 | 40 |
| **5 years** | 1.13 | (0.96 - 1.32) | 0.133 | 371 | 1.57 | (0.99 - 2.47) | 0.053 | 34 |
| **8 years** | 1.19 | (1.02 - 1.38) | 0.025 | 378 | 1.29 | (0.86 - 1.94) | 0.223 | 37 |
| **11 years** | 1.06 | (0.90 - 1.24) | 0.481 | 333 | 1.36 | (0.89 - 2.08) | 0.159 | 30 |
| **16 years** | 1.07 | (0.92 - 1.24) | 0.392 | 274 | 1.05 | (0.60 - 1.85) | 0.863 | 24 |
| **GEE: age 1-16** | 1.11 | (0.99 - 1.25) | 0.064 | 409 | 1.31 | (0.96 - 1.80) | 0.091 | 41 |
| **GEE: complete age 3-16** | 1.08 | (0.94 - 1.25) | 0.257 | 217 | 1.16 | (0.67 - 2.00) | 0.592 | 15 |
| ***Can f 1 exposure and dog sensitisation*** | | | | | | | | |
| **1 year** | 1.29 | (0.73 - 2.29) | 0.378 | 180 | 1.39 | (0.44 - 4.37) | 0.570 | 16 |
| **3 years** | 1.19 | (0.99 - 1.43) | 0.068 | 367 | 1.20 | (0.68 - 2.13) | 0.535 | 39 |
| **5 years** | 1.11 | (0.91 - 1.36) | 0.295 | 363 | 1.31 | (0.81 - 2.10) | 0.269 | 33 |
| **8 years** | 1.19 | (0.92 - 1.54) | 0.191 | 370 | 0.99 | (0.33 - 2.96) | 0.990 | 36 |
| **11 years** | 1.22 | (0.97 - 1.55) | 0.093 | 328 | 0.60 | (0.04 - 9.42) | 0.717 | 29 |
| **16 years** | 1.26 | (1.06 - 1.50) | 0.008 | 271 | 1.06 | (0.46 - 2.44) | 0.894 | 23 |
| **GEE: age 1-16** | 1.20 | (1.06 - 1.37) | 0.004 | 398 | 1.10 | (0.70 - 1.73) | 0.688 | 40 |
| **GEE: complete age 3-16** | 1.14 | (0.98 - 1.33) | 0.099 | 217 | 1.60 | (0.84 - 3.05) | 0.154 | 14 |

**Table E4:** Adjusted cross-sectional analysis showing the effect of *FLG* mutations and cat ownership on the risk of cat-specific sensitization. The GEE model: age 3-16. Ownership refers to the presence of a cat in the first year of life.

|  | ***FLG* Mutation, No Cat Present** | | |  | ***FLG* Wild Type, Cat Present** | | | ***FLG* Mutation, Cat Present** | | |
| --- | --- | --- | --- | --- | --- | --- | --- | --- | --- | --- |
|  | N | Odds Ratio (95% CI) | p-value | | n | Odds Ratio (95% CI) | p-value | n | Odds Ratio (95% CI) | p-value |
| **1 year** | 15 | n/a (small sample size) |  | | 56 | 0.60 (0.06 - 6.03) | 0.664 | 6 | n/a (small sample size) |  |
| **3 years** | 43 | 2.12 (0.69 - 6.55) | 0.191 | | 124 | 1.43 (0.61 - 3.34) | 0.410 | 18 | 4.14 (1.10 - 15.52) | 0.035 |
| **5 years** | 41 | 1.70 (0.67 - 4.28) | 0.260 | | 131 | 0.91 (0.45 - 1.82) | 0.791 | 17 | 4.13 (1.38 - 12.30) | 0.011 |
| **8 years** | 41 | 1.53 (0.61 - 3.86) | 0.359 | | 134 | 0.72 (0.35 - 1.48) | 0.377 | 18 | 4.48 (1.60 - 12.53) | 0.004 |
| **11 years** | 38 | 1.65 (0.65 - 4.17) | 0.293 | | 111 | 0.68 (0.31 - 1.50) | 0.341 | 14 | 2.39 (0.64 - 8.94) | 0.194 |
| **16 years** | 25 | 0.85 (0.28 – 2.56) | 0.766 | | 92 | 1.08 (0.60 - 1.95) | 0.799 | 10 | 1.11 (0.23 – 5.37) | 0.897 |
| **GEE** | 48 | 1.25 (0.60 - 2.58) | 0.550 | | 149 | 0.90 (0.55 - 1.49) | 0.692 | 19 | 3.02 (1.26 - 7.21) | 0.013 |

**Table E5:** Adjusted GEE analyses showing the interaction effect of cat ownership and *FLG* loss-of function mutation on the development of cat sensitisation from age 3 to 16.

Cat ownership refers to the presence of a cat in the first year of life

|  | **Sensitization to cat (n=483)** | |
| --- | --- | --- |
|  | OR (95% CI) | p-value |
| Cat present (1st year of life) | 0.90 (0.48 - 1.67) | 0.731 |
| *FLG* loss-of function mutation | 1.36 (0.53 - 3.45) | 0.520 |
| Cat present * *FLG* mutations | 2.89 (0.69 - 12.04) | 0.145 |
| Age | 1.08 (1.05 - 1.11) | <0.001 |
| Male | 1.54 (0.95 - 2.52) | 0.083 |
| Breast-fed ever | 1.12 (0.63 - 1.98) | 0.701 |
| Socio-economic status (managerial level) | 1.16 (0.68 - 1.96) | 0.590 |

**Table E6:** Adjusted cross-sectional analysis showing the effect of *FLG* mutations and dog ownership on the risk of dog-specific sensitization. The GEE model: age 3-16. Ownership refers to the presence of a cat in the first year of life.

*n/a: small sample size; no sensitized children among dog owners with FLG loss-of-function mutations

|  | ***FLG* Mutation, No Dog Present** | | |  | ***FLG* Wild Type, Dog Present** | | | ***FLG* Mutation, Dog Present** | | |
| --- | --- | --- | --- | --- | --- | --- | --- | --- | --- | --- |
|  | N | Odds Ratio (95% CI) | p-value | | n | Odds Ratio (95% CI) | p-value | n | Odds Ratio (95% CI) | p-value |
| **1 year** | 15 | 7.18 (0.61 – 84.09) | 0.006 | | 46 | 2.23 (0.20 – 25.17) | 0.516 | 6 | n/a* |  |
| **3 years** | 50 | 4.37 (1.79 – 10.66) | 0.001 | | 108 | 1.84 (0.78 – 4.34) | 0.167 | 11 | n/a* |  |
| **5 years** | 47 | 2.18 (0.79 – 6.02) | 0.131 | | 104 | 1.32 (0.55 – 3.16) | 0.528 | 11 | 4.07 (0.83 – 19.9) | 0.083 |
| **8 years** | 47 | 0.97 (0.12 – 7.71) | 0.973 | | 109 | 2.13 (0.71 – 6.38) | 0.175 | 12 | n/a* |  |
| **11 years** | 38 | 1.59 (0.35 – 7.28) | 0.549 | | 93 | 1.98 (0.74 – 5.28) | 0.175 | 12 | n/a* |  |
| **16 years** | 28 | 0.92 (0.26 – 3.18) | 0.890 | | 73 | 1.35 (0.66 – 2.80) | 0.413 | 7 | n/a* |  |
| **GEE** | 54 | 1.74 (0.81 – 3.71) | 0.153 | | 120 | 1.65 (0.95 2.86) | 0.076 | 13 | 0.63 (0.07 – 5.67) | 0.680 |

**Table E7:** Adjusted GEE analyses showing the interaction effect of dog ownership and *FLG* loss-of function mutation on the development of dog sensitisation from age 3 to 16.

Dog ownership refers to the presence of a dog in the first year of life

|  | **Sensitization to dog (n=483)** | |
| --- | --- | --- |
|  | Odds Ratio (95% CI) | p-value |
| Dog present (1st year of life) | 2.38 (1.21 - 4.67) | 0.012 |
| *FLG* loss-of function mutation | 2.97 (1.24 - 7.12) | 0.014 |
| Dog present * *FLG* mutations | 0.06 (0.001 - 1.72) | 0.100 |
| Age | 1.05 (1.00 - 1.09) | 0.031 |
| Male | 2.05 (1.09 - 3.88) | 0.027 |
| Breast-fed ever | 0.83 (0.42 - 1.66) | 0.601 |
| Socio-economic status (managerial level) | 1.43 (0.74 - 2.79) | 0.289 |

**Table E8:** Adjusted GEE analyses showing the interaction effect of cat ownership and *FLG* loss-of function mutation on the development of allergic sensitisation from age 3 to 16.

Cat ownership refers to the presence of a cat in the first year of life. Sensitization is defined as at least one positive test result to *Dermatophagoides pteronyssinus*, cat, dog, grass pollen, molds, milk, and egg (ages 1-5), birch and peanut (ages 8-16; ie. a total of 9 allergens)

|  | **Allergic sensitization (n=483)** | |
| --- | --- | --- |
|  | Odds Ratio (95% CI) | p-value |
| Cat present (1st year of life) | 1.07 (0.68 - 1.67) | 0.778 |
| *FLG* loss-of function mutation | 1.46 (0.71 - 3.01) | 0.307 |
| Cat present * *FLG* mutations | 1.41 (0.42 - 4.71) | 0.580 |
| Age | 1.08 (1.06 - 1.10) | 0.000 |
| Male | 1.84 (1.27 - 2.67) | 0.001 |
| Breast-fed ever | 1.09 (0.71 - 1.68) | 0.691 |
| Socio-economic status (managerial level) | 1.19 (0.80 - 1.77) | 0.385 |

**REFERENCES**

1. Luczynska CM, Arruda LK, Platts-Mills TA, Miller JD, Lopez M, Chapman MD. A two-site monoclonal antibody ELISA for the quantification of the major Dermatophagoides spp. allergens, Der p I and Der f I. J Immunol Methods. 1989;118(2):227-35.

2. Custovic A, Simpson A, Pahdi H, Green RM, Chapman MD, Woodcock A. Distribution, aerodynamic characteristics, and removal of the major cat allergen Fel d 1 in British homes. Thorax. 1998;53(1):33-8.

3. Custovic A, Green R, Fletcher A, Smith A, Pickering CAC, Chapman MD, et al. Aerodynamic properties of the major dog allergen Can f 1: Distribution in homes, concentration, and particle size of allergen in the air. American journal of respiratory and critical care medicine. 1997;155(1):94-8.
